# Associations of weight-adjusted-waist index and depression with secondary infertility

**DOI:** 10.3389/fendo.2024.1330206

**Published:** 2024-03-07

**Authors:** Fei Sun, Min Liu, Shanshan Hu, Ruijie Xie, Huijuan Chen, Zhaona Sun, Huiya Bi

**Affiliations:** ^1^ Wuxi Medical College of Jiangnan University, Wuxi, China; ^2^ Department of Nursing, Wuxi Maternity and Child Health Care Hospital, Wuxi, China; ^3^ Department of Microsurgery, University of South China, Hengyang, China

**Keywords:** secondary infertility, weight-adjusted-waist index (WWI), depression, NHANES, ROC, mediating effect

## Abstract

**Background:**

Obesity and psychological factors were identified as risk factors for female infertility. The study investigated the correlation between WWI, depression, and secondary infertility, focusing on the potential mediating role of depression.

**Methods:**

According to the data from NHANES, this cross-sectional study used multiple regression analysis, subgroup analysis, and smooth curve fitting to explore the relationship between WWI, depression, and secondary infertility. The diagnostic ability of WWI was evaluated and compared to other obesity indicators using the ROC curve. The mediating effect test adopted the distribution of the product.

**Results:**

This study involved 2778 participants, including 381 (13.7%) women with secondary infertility. Results showed that higher WWI (OR = 1.31; 95% CI, 1.11-1.56) and depression scores (OR = 1.03; 95% CI, 1.01-1.06) were associated with secondary infertility. There was a positive correlation between WWI and secondary infertility (nonlinear *p* = 0.8272) and this association was still consistent in subgroups (all *P* for interaction> 0.05). Compared with other obesity indicators, WWI (AUC = 0.588) also shows good predictive performance for secondary infertility. Mediation analysis showed that depression mediated the relationship between 3.94% of WWI and secondary infertility, with a confidence interval of Z_a_ * Z_b_ excluding 0.

**Conclusion:**

WWI exhibited a relatively good correlation in predicting secondary infertility than other obesity indicators, and depression may be a mediator between WWI and secondary infertility. Focusing on the potential mediating role of depression, the risk of secondary infertility due to obesity may be beneficially reduced in women.

## Introduction

Secondary infertility refers to a woman who has previously been pregnant and has had regular sexual activity without contraception, but has not been pregnant for 12 consecutive months (1). Infertility troubles thousands of women of childbearing age, and according to statistics, the incidence of infertility ranges from 3% to 30% (2, 3). A multicenter study in China shows that 24.58% of women have infertility, and the incidence of secondary infertility is 18.04% (4). The factors that cause female infertility include reproductive system-related diseases, abnormal immune function, and social and physiological psychological factors (5, 6). More and more evidence also emphasizes the impact of lifestyle on infertility (7). A comprehensive understanding of easily modifiable risk factors, such as obesity and depression, is crucial for preventing and controlling secondary infertility in women.

Obese women exhibit a significantly increased risk of anovulatory infertility (8). Especially central obesity and visceral fat can lead to “hyperinsulinemia”. “Insulin resistance” directly affects the ovaries, reducing the production of liver sex hormone binding globulin, leading to hyperandrogenism (9, 10). These changes all disrupt the hypothalamic-pituitary gonadal axis, decreasing fertility (11). Currently, most studies use body mass index (BMI) and a body shape index (ABSI) to evaluate overall and abdominal fat content (12, 13). In contrast, the weight-adjusted-waist index (WWI) is more sensitive in assessing central obesity and predicting female infertility (14). The WWI is an anthropometric measure of central obesity, defined as WC divided by the square of body weight. It can reflect the composition of fat and muscle mass, even in different BMI categories (15). Studies have shown that WWI levels were positively linked to an increased risk of infertility in American females and showed a stronger association than other markers of obesity (14). Although WWI as a central obesity indicator can effectively predict infertility (16), the relationship between WWI and secondary infertility in women has not been studied before.

At present, studies have also shown a positive correlation between WWI and depressive symptoms, and this association is stronger than BMI and waist circumference (17). Depression plays an crucial mediating role in many diseases, including inflammatory bowel disease and breast cancer (18, 19). However, there are few reports on the psychological mechanisms between WWI and secondary infertility. There is a bidirectional connection between depression and infertility; that is, depression is a risk factor for infertility and a common psychological symptom in infertile women (20, 21). Compared to women with primary infertility, women with secondary infertility have a higher probability of psychological distress (22). Women with depression are less likely to use healthcare to treat infertility (23). Over time, changes in endocrine hormones and emotional disorders may affect the growth of the endometrium making it difficult to effectively address secondary infertility (24, 25). Given the relationship between WWI, depression, and infertility, depression may play a role in the association between WWI and secondary infertility.

Therefore, the study investigates the relationship between WWI and depression in women aged 18-45 in the United States and secondary infertility. To our knowledge, this is the first study to evaluate the relationship between WWI and secondary infertility. In addition, this study hypothesizes that depression plays a mediating role in the relationship between WWI and secondary infertility. On the basis of exploring the relationship between WWI, depression, and secondary infertility, analyze the role of depression in the relationship between WWI and secondary infertility.

## Materials and methods

### Survey description

The cross-sectional data was from NHANES, a national study conducted by the National Center for Health Statistics (NCHS) to evaluate the nutritional and health status of the United States. The Research Ethics Review Committee of NCHS approved all NHANES research protocols and obtained written informed consent from all survey participants. Participants provided demographic, dietary, examination, laboratory and questionnaire data. All detailed NHANES research designs and data are publicly available at www.cdc.gov/nchs/NHANEs/. This study followed the Strengthening the Reporting of Observational Studies in Epidemiology (STROBE) reporting guidelines for cross-sectional studies.

### Study population

**Figure 1:**
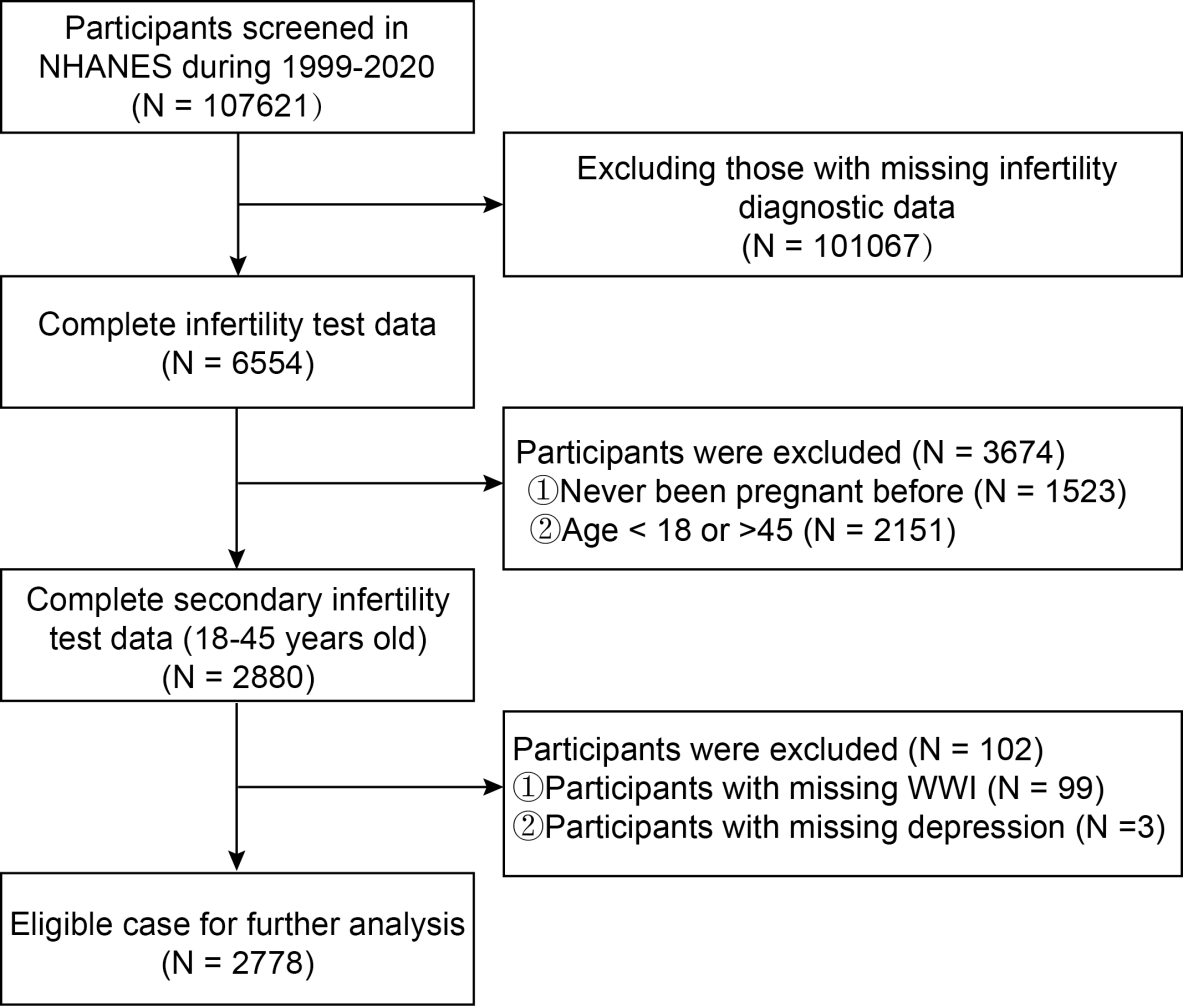
Flowchart of study population inclusion in 1999–2020 NHANES. NHANES, National Health and Nutrition Examination Survey; WWI, weight-adjusted-waist index.

The NHANES datasets were utilized for this investigation from 1999-2020. Participants with complete data on WWI, depression and secondary infertility participated in our analysis. Initially, a total of 107,621 participants were recruited. They were excluding those with missing secondary infertility diagnostic (n=101067), age not between 18 and 45 years old (n=2151), never been pregnant before (n=1523), missing data about depression (n=3), and WWI (n=99). The missing covariate values were represented by means or other. Our final analysis included 2778 eligible participants (**Figure 1**).

### Assessment of secondary infertility

Secondary infertility was determined based on the Reproductive Health Questionnaire (RHQ) questions RHQ074: “Tried for a year to become pregnant?” and RHQ131”Ever been pregnant?”. Participants who answered “yes” to the two questions were considered as having secondary infertility.

### Assessment of weight-adjusted-waist index

WWI samples of NHANES participants were obtained in examination data. The WWI calculation was based on the participant’s waist circumference divided by the square root of their weight (26). The unit of waist circumference is centimeters, and the unit of weight is kilograms. The WWI score is positively correlated with central obesity (27) (Equation 8). WWI was treated as a continuous variable and designed as an exposed variable in our study.

### Assessment of other obesity indicators

Typical obesity indicators include waist circumference (WC), body mass index (BMI) (Equation 1), waist-BMI ratio (wBMI) (Equation 4), waist-to-height ratio (WHtR) (Equation 2), waist-to-hip ratio (WHR) (Equation 3), conicity index (CI) (Equation 5), body roundness index (BRI) (Equation 7), and a body shape index (ABSI) (Equation 6). These obesity indicators were determined based on the Body Measures Examination (BMX) items. The calculation formula for these indicators is as follows (13, 14):


(1) $$BMI=\frac{Weight(kg)}{Height{\left(m\right)}^{2}}$$

(2) $$WHtR=\frac{WC(cm)}{Height(cm)}$$

(3) $$WHR=\frac{WC(cm)}{Hip(cm)}$$

(4) wBMI=WC(m)∗BMI

(5) $$CI=\frac{WC(m)}{0.109\sqrt{\frac{Weight(kg)}{Height(m)}}}$$

(6) $$ABSI=\frac{WC(m)}{BMI^{2/3}*Height{\left(m\right)}^{1/2}}$$

(7) $$BRI=364.2-365.5\sqrt{1-\{\frac{{\left[WC(m)/2\pi \right]}^{2}}{{\left[0.5*Height(m)\right]}^{2}}}\}$$

(8) $$WWI=\frac{WC(cm)}{\sqrt{Weight(kg)}}$$

### Assessment of depression

Depression samples of NHANES participants were obtained in questionnaire data. The Patient Health Questionnaire-9 (PHQ-9) has 9 items and is a simple and effective self-assessment scale for depression disorders, with good reliability and validity, as demonstrated by a reported Cronbach’ s α 0.89 (28, 29). A score of 0-4 indicates no depression, a score of 5-9 indicates mild depression, a score of 10-14 indicates moderate depression, and a score of ≥ 15 indicates severe depression (29).

### Assessment of covariates of interest

Demographic and dietary covariates in our study included age, race, education level, marital status, family income to poverty ratio, and dietary fiber (gm). Body measure covariates included BMI. The questionnaire variates composed of alcohol use, diabetes, sleep disorders, minutes of sedentary activity, age when first menstrual period occurred, had regular periods in past 12 months, ever treated for a pelvic infection/PID and ever taken birth control pills were also included. All detailed measurement processes of these variables are publicly available at www.cdc.gov/nchs/nhanes/.

### Statistical analysis

Data description and statistical analysis were based on complex weighting in descriptive analysis. Continuous variables were summarized as the means with standard error (SE), and categorical parameters were presented as proportions. Using logistic regression analysis to analyze the risk relationship between WWI and secondary infertility, this study constructed three logistic regression models that adjusted for different confounding factors. Among them, Model 1: did not adjust for any confounding factors; Model 2: adjusted for age, and race; Model 3: further adjustments were made to age, ethnicity/race, education level, marital status, family income, BMI, alcohol use, diabetes, dietary fiber (gm), minutes sedentary activity, sleep disorder, age when first menstrual period occurred, had regular periods in past 12 months, ever treated for a pelvic infection/PID and ever taken birth control pills based on Model 2. In sensitivity analysis, linear trend tests were conducted with WWI quartile groups as independent variables to evaluate its robustness (30). Subgroup analysis was performed using a stratified multivariable logistic regression model, and stratified factors included age, ethnicity/race, education level, marital status, BMI, diabetes, PHQ-9 score, had regular periods in past 12 months, ever treated for a pelvic infection/PID, and ever taken birth control pills. This study used smooth curve fitting to observe whether the relationship between WWI and secondary infertility is linear. If non-linear, threshold effect analysis was used to provide each interval and calculate the threshold. We also analyzed the ability of WWI and other obesity indicators to predict secondary infertility through the receiver operating characteristic (ROC) curve and the area under the comparison curve (AUC) value. Finally, the product distribution method was used to test the mediating effect of depression between WWI and secondary infertility (31, 32). Other representations were used for missing values in classification variables based on existing data, while for missing values in continuous variables, mean interpolation was used. PackageR (http://www.r-project.org) and EmpowerStats (http://www.empowerstats.com) were used for all analyses. Statistical significance was assessed at a two-sided value of *P*<0.05.

## Results

### Baseline characteristics of participants

A total of 2778 secondary infertility participants were selected, all of whom were women aged 18-45. **Table 1** presents the characteristics of the NHANES participants by secondary infertility status. Among these, 381 females were diagnosed with secondary infertility, accounting for 13.71% of participants. Females with secondary infertility were older (35.72 ± 6.54 years old), had higher BMI, and family income and education level, and had a higher proportion of diabetes and sleep disorders than those without secondary infertility. The secondary infertility group tended to be non-Hispanic white race/ethnicity and never married more than the healthy controls. What’s more, secondary infertility females demonstrated higher PHQ-9 scores and WWI.

**Table 1:** Characteristics of the study population in NHANES by secondary infertility status.

Variable	Total (N = 2778)	Fertile (N = 2397)	Infertile (N = 381)	OR 95% CI	*P*-value
Age, years	34.65 ± 7.00	34.48 ± 7.06	35.72 ± 6.54	1.03 (1.01, 1.04)	0.0014
Ethnicity, %					0.005
Mexican American	486 (17.49%)	432 (18.02%)	54 (14.17%)	1.0 (Reference)	
Other Hispanic	307 (11.05%)	266 (11.10%)	41 (10.76%)	1.23 (0.80, 1.90)	0.3438
Non-Hispanic White	854 (30.74%)	708 (29.54%)	146 (38.32%)	1.65 (1.18, 2.30)	0.0033
vNon-Hispanic Black	710 (25.56%)	630 (26.28%)	80 (21.00%)	1.02 (0.70, 1.47)	0.9328
Other Race	421 (15.15%)	361 (15.06%)	60 (15.75%)	1.33 (0.90, 1.97)	0.1557
Family income, %					<0.001
0 to 4.99	2225 (80.09%)	1936 (80.77%)	289 (75.85%)	1.0 (Reference)	
≥5	302 (10.87%)	238 (9.93%)	64 (16.80%)	1.80 (1.33, 2.44)	0.0001
Other	251 (9.04%)	223 (9.30%)	28 (7.35%)	0.84 (0.56, 1.27)	0.4104
Education level, %					<0.001
Less than 9th grade	169 (6.08%)	155 (6.47%)	14 (3.67%)	1.0 (Reference)	
9-11th grade	362 (13.03%)	324 (13.52%)	38 (9.97%)	1.30 (0.68, 2.47)	0.4250
High school graduate/GED or equivalent	574 (20.66%)	510 (21.28%)	64 (16.80%)	1.39 (0.76, 2.55)	0.2870
Some college or AA degree	1037 (37.33%)	884 (36.88%)	153 (40.16%)	1.92 (1.08, 3.40)	0.0261
College graduate or above	636 (22.89%)	524 (21.86%)	112 (29.40%)	2.37 (1.32, 4.24)	0.0038
Marital Status, %					<0.001
Married/Living with Partner	1814 (65.30%)	1531 (63.87%)	283 (74.28%)	1.0 (Reference)	
Widowed/Divorced/Separated	225 (8.10%)	198 (8.26%)	27 (7.09%)	0.74 (0.48, 1.12)	0.1573
Never married	739 (26.60%)	668 (27.87%)	71 (18.64%)	0.58 (0.44, 0.76)	<0.0001
Alcohol use, %					0.109
No	301 (10.84%)	255 (10.64%)	46 (12.07%)	1.0 (Reference)	
Yes	2052 (73.87%)	1762 (73.51%)	290 (76.12%)	0.91 (0.65, 1.28)	0.5945
Other	425 (15.30%)	380 (15.85%)	45 (11.81%)	0.66 (0.42, 1.02)	0.0611
BMI (kg/m^2^), %					<0.001
Normal weight<25	808 (29.09%)	703 (29.33%)	105 (27.56%)	1.0 (Reference)	
Overweight [25, 30)	708 (25.49%)	636 (26.53%)	72 (18.90%)	0.76 (0.55, 1.04)	0.0881
Obese ≥30	1262 (45.43%)	1058 (44.14%)	204 (53.54%)	1.29 (1.00, 1.66)	0.0488
Diabetes, %					0.013
Yes	146 (5.26%)	114 (4.76%)	32 (8.40%)	1.0 (Reference)	
No	2586 (93.09%)	2243 (93.58%)	343 (90.03%)	0.54 (0.36, 0.82)	0.0035
Borderline	46 (1.66%)	40 (1.67%)	6 (1.57%)	0.53 (0.21, 1.37)	0.1929
Sleep disorder					0.024
No	871 (31.35%)	767 (32.00%)	104 (27.30%)	1.0 (Reference)	
Yes	302 (10.87%)	247 (10.30%)	55 (14.44%)	1.64 (1.15, 2.35)	0.0064
Other	1605 (57.78%)	1383 (57.70%)	222 (58.27%)	1.18 (0.92, 1.52)	0.1841
Age when first menstrual period occurred					0.831
10-12 years	9 (0.32%)	8 (0.33%)	1 (0.26%)	1.0 (Reference)	
13-15 years	2 (0.07%)	2 (0.08%)	0 (0.00%)	0.00 (0.00, Inf)	0.9758
Other	2767 (99.60%)	2387 (99.58%)	380 (99.74%)	1.27 (0.16, 10.21)	0.8199
Had regular periods in past 12 months					0.791
Yes	2431 (87.51%)	2096 (87.44%)	335 (87.93%)	1.0 (Reference)	
No	347 (12.49%)	301 (12.56%)	46 (12.07%)	0.96 (0.69, 1.33)	0.7907
Ever treated for a pelvic infection/PID					0.002
Yes	167 (6.01%)	131 (5.47%)	36 (9.45%)	1.0 (Reference)	
No	2590 (93.23%)	2245 (93.66%)	345 (90.55%)	0.56 (0.38, 0.82)	0.0032
Other	21 (0.76%)	21 (0.88%)	0 (0.00%)	0.00 (0.00, inf.)	0.9642
Ever taken birth control pills					0.001
Yes	1102 (39.67%)	926 (38.63%)	176 (46.19%)	1.0 (Reference)	
No	501 (18.03%)	455 (18.98%)	46 (12.07%)	0.53 (0.38, 0.75)	0.0003
Other	1175 (42.30%)	1016 (42.39%)	159 (41.73%)	0.82 (0.65, 1.04)	0.1009
Dietary fiber (gm)	14.89 ± 8.02	14.95 ± 8.05	14.47 ± 7.81	0.99 (0.98, 1.01)	0.2804
PHQ-9 score	3.70 ± 4.49	3.59 ± 4.45	4.35 ± 4.71	1.03 (1.01, 1.06)	0.0025
Minutes sedentary activity	348.16 ± 203.72	345.55 ± 202.69	364.60 ± 209.60	1.00 (1.00, 1.00)	0.0901
WC	98.01 ± 18.18	97.30 ± 17.76	102.49 ± 20.11	1.02 (1.01, 1.02)	><0.001
WHtR	0.61 ± 0.11	0.60 ± 0.11	0.63 ± 0.12	8.49 (3.33, 21.67)	<0.001
wBMI	31.13 ± 14.65	30.62 ± 14.20	34.31 ± 16.90	1.02 (1.01, 1.02)	<0.001
CI	1.29 ± 0.09	1.29 ± 0.09	1.31 ± 0.09	26.94 (8.22, 88.30)	<0.001
BRI	5.84 ± 2.72	5.74 ± 2.65	6.44 ± 3.09	1.09 (1.05, 1.13)	<0.001
ABSI	0.08 ± 0.01	0.08 ± 0.01	0.08 ± 0.01	0.22 (0.11, 0.33)	<0.001
WWI	11.06 ± 0.80	11.04 ± 0.79	11.23 ± 0.82	1.35 (1.18, 1.54)	<0.001

“Inf” represents infinity and belongs to the floating-point type. It is generally infinite when the divisor is 0.

PHQ-9, Patient Health Questionnaire 9; OR, odds ratio; CI, confidence interval; WC, waist circumference; BMI, body mass index; wBMI, waist-BMI ratio; WHtR, waist-to-height ratio; CI, Conicity index; BRI, body roundness index; ABSI, a body shape index; WWI,weight-adjusted-waist index; Family income (%), the ratio of family income to poverty.

### The associations of weight-adjusted-waist index and depression with secondary infertility

Logistic regression analysis showed that after adjusting for all confounding factors, the correlation between WWI, depression, and secondary infertility remained significant. As shown in **Table 2**, an increase of one unit in WWI is associated with a 31% increase in the prevalence of secondary infertility, while an increase of one point in the PHQ-9 score is associated with a 3% increase in the prevalence of secondary infertility. Sensitivity analysis using WWI as a categorical variable (quartile) still showed significant statistical significance (OR=1.33, 95% CI: 1.09-1.62; *P* = 0.0044). Subgroup analysis for the association between WWI and infertility (**Table 3**). None of the stratifications including age, ethnicity, education lever, marital status, PHQ-9 score, BMI, diabetes, had regular periods in past 12 months, ever treated for a pelvic infection/PID and ever taken birth control pills affected the positive association of WWI and infertility

**Table 2:** Association between weight-adjusted-waist index and secondary infertility.

Variable	Model 1		Model 2		Model 3	
	*OR* (95% CI)	*P-*value	*OR* (95% CI)	*P-*value	*OR* (95% CI)	*P-*value
WWI	1.35 (1.18, 1.54)	<0.0001	1.36 (1.18, 1.56)	<0.0001	1.31 (1.11, 1.56)	0.0018
Quartiles of WWI						
Q1	1.0 (Reference)		1.0 (Reference)		1.0 (Reference)	
Q2	1.19 (0.85, 1.66)	0.3064	1.17 (0.84, 1.64)	0.3481	1.10 (0.78, 1.55)	0.5974
Q3	1.45 (1.05, 2.00)	0.0232	1.49 (1.08, 2.07)	0.0167	1.42 (1.00, 2.01)	0.0518
Q4	1.81 (1.33, 2.48)	0.0002	1.85 (1.35, 2.54)	0.0001	1.65 (1.13, 2.40)	0.0097
P for trend	1.39 (1.18, 1.63)	<0.0001	1.41 (1.20, 1.65)	<0.0001	1.33 (1.09, 1.62)	0.0044
PHQ-9 score	1.03 (1.01, 1.06)	0.0025	1.03 (1.01, 1.06)	0.0030	1.03 (1.01, 1.06)	0.0083
Severity of depression						
0-4	1.0 (Reference)		1.0 (Reference)		1.0 (Reference)	
5-9	1.39 (1.06, 1.82)	0.0159	1.43 (1.09, 1.88)	0.0100	1.41 (1.06, 1.87)	0.0170
10-14	1.60 (1.08, 2.37)	0.0181	1.61 (1.09, 2.39)	0.0172	1.74 (1.15, 2.64)	0.0085
≥15	1.32 (0.77, 2.25)	0.3132	1.25 (0.73, 2.14)	0.4193	1.14 (0.64, 2.03)	0.6654

Insensitivity analysis, the weight-adjusted-waist index was converted from a continuous variable to a categorical variable (quartiles).

Abbreviations: WWI,weight-adjusted-waist index; BMI, body mass index, PHQ-9, Patient Health Questionnaire 9; OR, odds ratio; CI, confidence interval.

Model 1: no covariates were adjusted.

Model 2: age and ethnicity were adjusted.

Model 3: age, ethnicity, education level, marital status, family income, BMI, alcohol use, diabetes, dietary fiber (gm), minutes sedentary activity, sleep disorder, PHQ-9 score, age when first menstrual period occurred, had regular periods in past 12 months, ever treated for a pelvic infection/PID and ever taken birth control pills were adjusted.

**Table 3:** Subgroups analyses of the effect of WWI on secondary infertility.

Subgroups	N		*OR* (95% CI), *P-*value	*P* for interaction
	Total	Secondary infertility		
Age				0.2618
Tertile 1	828	87 (22.83%)	1.57 (1.20, 2.06) 0.0009	
Tertile 2	959	143 (37.53%)	1.35 (1.08, 1.69) 0.0092	
Tertile 3	991	151 (39.63%)	1.18 (0.95, 1.47) 0.1436	
Ethnicity, %				0.9026
Mexican American	486	54 (14.17%)	1.47 (0.98, 2.21) 0.0659	
Other Hispanic	307	41 (10.76%)	1.52 (1.00, 2.32) 0.0523	
Non-Hispanic White	854	146 (38.32%)	1.28 (1.03, 1.59) 0.0248	
Non-Hispanic Black	710	80 (21.00%)	1.47 (1.13, 1.92) 0.0047	
Other Race	421	60 (15.75%)	1.29 (0.89, 1.87) 0.1730	
Education level, %				0.2620
Less than 9th grade	169	14 (3.67%)	1.11 (0.55, 2.22) 0.7672	
9-11th grade	362	38 (9.97%)	2.33 (1.45, 3.73) 0.0004	
High school graduate/GED or equivalent	574	64 (16.80%)	1.29 (0.93, 1.78) 0.1281	
Some college or AA degree	1037	153 (40.16%)	1.42 (1.16, 1.76) 0.0009	
College graduate or above	636	112 (29.40%)	1.39 (1.05, 1.83) 0.0196	
Marital Status, %				0.2585
Married/Living with Partner	1814	283 (74.28%)	1.29 (1.09, 1.52) 0.0023	
Widowed/Divorced/Separated	225	27 (7.09%)	1.05 (0.63, 1.75) 0.8634	
Never married	739	71 (18.64%)	1.60 (1.21, 2.12) 0.0009	
PHQ-9 score				0.4062
0-4	1972	245 (64.30%)	1.29 (1.09, 1.53) 0.0032	
5-9	509	84 (22.05%)	1.58 (1.18, 2.12) 0.0021	
10-14	189	35 (9.19%)	1.58 (1.18, 2.12) 0.0021	
≥15	108	17 (4.46%)	1.62 (0.83, 3.16) 0.1540	
BMI				0.4205
<25	808	105 (27.56%)	1.43 (1.03, 1.99) 0.0309	
[25, 30)	708	72 (18.90%)	1.08 (0.76, 1.52) 0.6759	
≥30	1262	204 (53.54%)	1.38 (1.12, 1.69) 0.0021	
Diabetes				0.8700
Yes	146	32 (8.40%)	1.40 (0.78, 2.52) 0.2657	
No	2586	343 (90.03%)	1.32 (1.14, 1.52) 0.0001	
Borderline	46	6 (1.57%)	0.99 (0.32, 3.09) 0.9892	
Had regular periods in past 12 months				0.8245
Yes	2431	335 (87.93%)	1.34 (1.16, 1.55)<0.0001	
No	347	46 (12.07%)	1.41 (0.96, 2.07) 0.0801	
Ever treated for a pelvic infection/PID				0.3007
Yes	167	36 (9.45%)	1.90 (1.19, 3.03) 0.0069	
No	2590	345 (90.55%)	1.30 (1.13, 1.50) 0.0003	
Ever taken birth control pills				0.3343
Yes	1102	176 (46.19%)	1.37 (1.12, 1.68) 0.0022	
No	501	46 (12.07%)	1.07 (0.73, 1.57) 0.7159	

WWI, weight-adjusted-waist index; BMI, body mass index, PHQ-9, Patient Health Questionnaire 9; OR, odds ratio; CI, confidence interval.

### WWI showed a good correlation than other markers of obesity including WC, BMI, WHtR, WHR, wBMI, BRI and ABSI for secondary infertility

**Figure 2:**
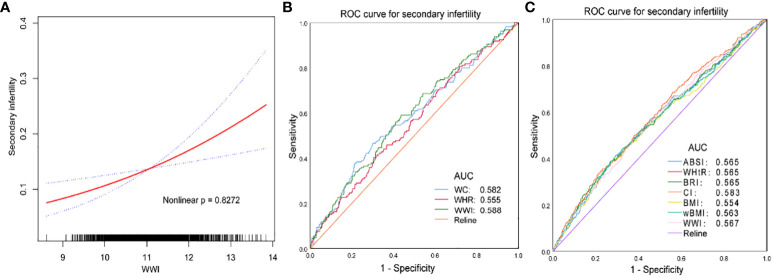
**(A, C)** the NHANES datasets were utilized for this investigation from 1999-2020. **(B)** the NHANES datasets were utilized for this investigation from 2017-2020. WC, waist circumference; BMI, body mass index; wBMI, waist-BMI ratio; WHtR, waist-to-height ratio; WHR, waist-to-hip ratio; CI, Conicity index; BRI, body roundness index; ABSI, a body shape index; WWI,weight-adjusted-waist index. Age, ethnicity, education level, marital status, family income, BMI, alcohol use, diabetes, dietary fiber (gm), minutes sedentary activity, sleep disorder, PHQ-9 score, age when first menstrual period occurred, had regular periods in past 12 months, ever treated for a pelvic infection/PID and ever taken birth control pills were adjusted.

Smooth curve fitting exhibited a positive relationship between WWI and secondary infertility (**Figure 2A**). Then, we calculated the AUC values to compare the predictive accuracy of WWI with other obesity indicators (WC, BMI, WHtR, WHR, wBMI, CI, BRI and ABSI) for secondary infertility (**Figures 2B, C**). We found that the AUC values of WWI and CI were higher than the other obesity indicators in predicting secondary infertility. Moreover, the difference in AUC values between WWI and other obesity indicators was statistically significant (all *P*< 0.05), suggesting that WWI maybe have a good advantage in evaluating secondary infertility (**Table 4**).

**Table 4:** Comparison of AUC values between WWI and other obesity indicators.

Test	AUC^1^	95%CI^2^	Best threshold	Sensitivity	Specificity	P for different in AUC
The NHANES datasets were utilized for this investigation from 1999-2020.						
WWI	0.567	0.536-0.598	11.269	0.483	0.627	< 0.001
BMI	0.554	0.522-0.587	33.550	0.396	0.719	0.001
ABSI	0.565	0.534-0.597	0.082	0.438	0.678	< 0.001
WHtR	0.565	0.534-0.597	0.665	0.386	0.733	< 0.001
BRI	0.565	0.534-0.597	6.982	0.386	0.733	< 0.001
CI	0.583	0.552-0.614	1.267	0.685	0.441	< 0.001
wBMI	0.563	0.530-0.595	34.741	0.415	0.705	< 0.001
The NHANES datasets were utilized for this investigation from 2017-2020.						
WWI	0.588	0.540-0.635	11.109	0.591	0.564	< 0.001
WC	0.582	0.533-0.631	105.650	0.465	0.700	0.001
WHR	0.555	0.507-0.604	0.762	0.403	0.696	0.025

^1^AUC, area under the curve; ^2^95% CI, 95% confidence interval; WC, waist circumference; BMI, body mass index; wBMI, waist-BMI ratio; WHtR, waist-to-height ratio; WHR, waist-to-hip ratio; CI, Conicity index; BRI, body roundness index; ABSI, a body shape index; WWI,weight-adjusted-waist index.

### Mediation analyses

The first step is to perform a logistic regression analysis of WWI(X) for secondary infertility(Y), and obtain c=0.323, *SE*(c)=0.072; Perform linear regression analysis of WWI(X) for depression(M), and obtain coefficients a=0.312 and *SE*(a)=0.108; Finally, a logistic regression analysis was conducted between WWI(X) and depression(M) for secondary infertility(Y), and the coefficients b=0.041, c ‘=0.310, *SE*(b)=0.012, and *SE*(c’) =0.073 were obtained (**Table 5**).

**Table 5:** Using the product distribution method to test the mediating effect.

Parameter		B/OR	S.E.	P	95% CI
X-Y(logistic regression)		0.323	0.072	< 0.001	1.199-1.592
X-M(linear regression)		0.312	0.108	0.004	0.101-0.523
X+M-Y(logistic regression)	X-Y	0.310	0.073	< 0.001	1.182-1.572
	M-Y	0.041	0.012	0.001	1.018-1.066

OR, odds ratio; 95% CI, 95% confidence interval; B, beta; S.E., standard error.

The regression coefficients of logistic regression results are interpreted using OR values, while the regression coefficients of linear regression results are interpreted using B.

X represents weight-adjusted-waist index(WWI), M represents depression, and Y represents secondary infertility.

Age, ethnicity, education level, marital status, family income, alcohol use, diabetes, dietary fiber (gm), minutes of sedentary activity, and sleep disorder were adjusted.

The second step is that the total effect (p<0.001) and direct effect(p<0.001) of WWI on secondary infertility are statistically significant. The 95% confidence interval for mediating effects obtained from the R Mediation software package of R software using the product distribution method is [2.390833, 21.252207], excluding 0. Therefore, the mediating effect of depression between WWI and secondary infertility is significant.

**Figure 3:**
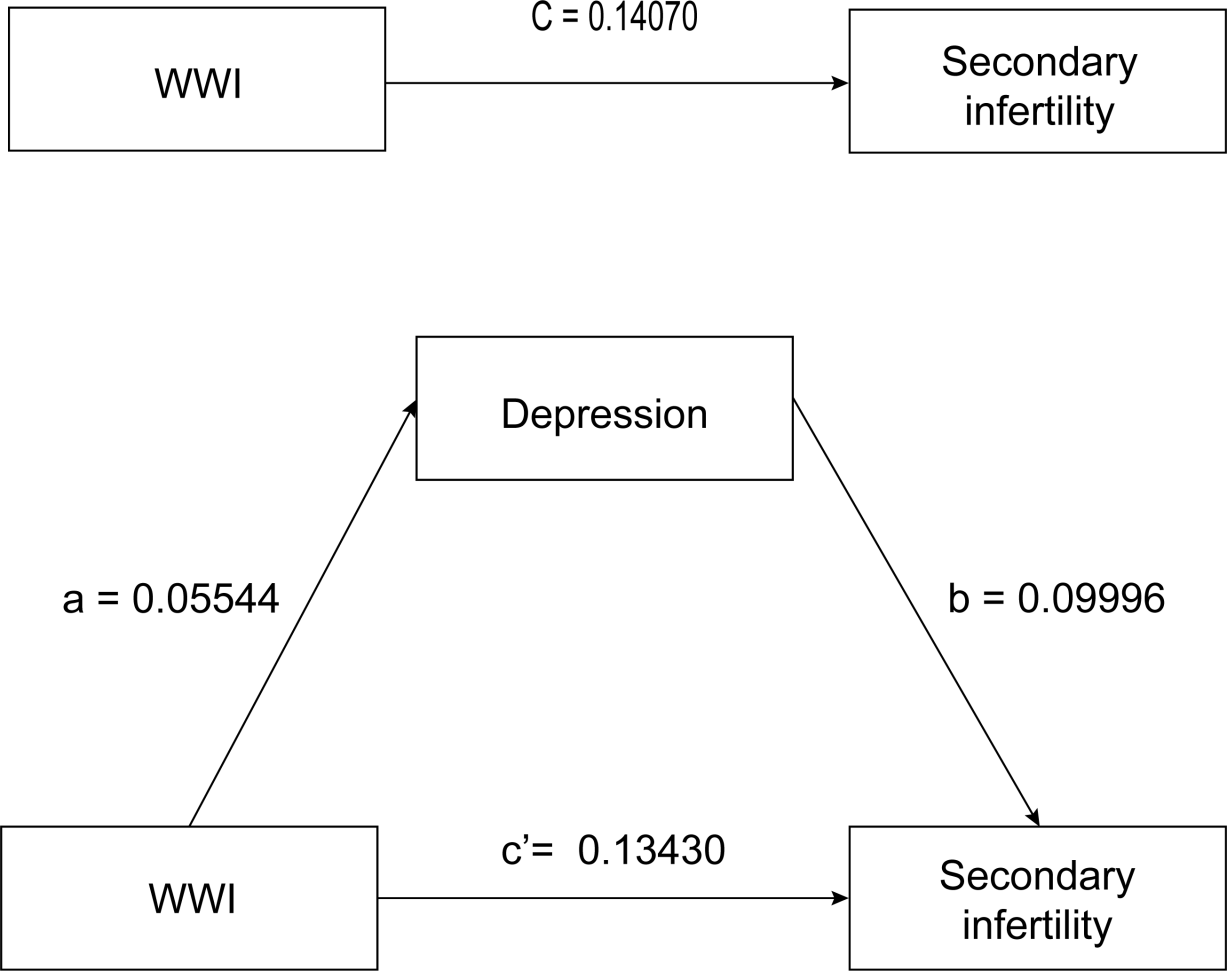
WWI, weight-adjusted-waist index. Age, ethnicity, education level, marital status, family income, alcohol use, diabetes, dietary fiber (gm), minutes sedentary activity, and sleep disorder were adjusted. Total effect 0.14070; Direct effect 0.13430; Indirect effect 0.00554.

Step 3, calculated by SPSS software, *SD*(X)=0.798, *SD*(M)=4.491, *Var*(X)=0.638, *Var*(M)=20.171, and *Cov*(X, M)=0.265. According to the formula, *Var*(Y’)=3.356, *Var*(Y’’)=3.392, then *SD*(Y’)=1.832, *SD*(Y ‘‘)=1.842. Calculate using the formula to obtain a_STD_=0.0554, b_STD_=0.09996, c’_STD_=0.13430, c_STD_=0.14070. Specific formula: a_STD_=a*SD*(X)/*SD*(M), b_STD_=b*SD*(M)/*SD*(Y’’), c_STD_ =c*SD*(X)/*SD*(Y’), c’_STD_=c’*SD*(X)/*SD*(Y’’), *Var*(Y’)=c^2^ *Var*(X)+π^2^/3, *Var*(Y’’)=c’^2^ *Var*(X)+b^2^ *Var*(M)+ 2bc’*Cov*(X,M)+π^2^/3. In the above formula, there is a subscript STD on the left, which represents the standardized coefficient converted from the coefficient in Logistic units. Where π^2^/3 is the variance of the standard logistic distribution. The mediating effect of depression accounts for 3.94% of the total effect [(a_STD_ × b_STD_/(a_STD_ × b_STD_+c’_STD_). The analysis of the mediating effect pathway is shown in **Figure 3**.

## Discussion

Secondary infertility accounts for a relatively high proportion of women among all infertility patients (2). This study investigated the relationship between WWI and depression in women aged 18-45 in the United States and secondary infertility. To our knowledge, this is the first study to evaluate the relationship between WWI and secondary infertility. We found a positive correlation between WWI and secondary infertility in rough and adjusted models. Sensitivity analysis using WWI as a quartile also revealed a correlation between WWI (OR=1.33; 95% CI, 1.09-1.62, P =0.0044)and secondary infertility. Subgroup analysis showed that this association between WWI and secondary infertility was stable across various demographic situations. The results of this study are similar to other studies, Wen et al. also found that WWI (OR=1.42, 95% CI: 1.22-1.65) hurts a person’s fertility (14). Legese et al. found that the incidence of secondary infertility is significantly higher in obese women (33). More and more evidence suggests that obesity can lead to anovulation, menstrual irregularities, reduced conception rates, and reduced response to fertility treatment (34, 35). However, the underlying causes of this unfavorable relationship between females’ secondary infertility and WWI are uncertain. It may be that adipose tissue affects the ovarian and endometrial function of obese women by producing leptin, free fatty acids, and cytokines (36).

We further found that compared to other obesity markers, the correlation between WWI, CI and secondary infertility is stronger, indicating that WWI may be more accurate in predicting secondary infertility than other obesity-related indicators. WWI is a newly developed obesity indicator that can more accurately predict total body fat percentage and has been explored in various fields (37, 38). Park et al. research shows that WWI is closely related to the incidence rate of cardiac metabolism and cardiovascular death, which is not apparent in BMI (39). Qin et al. observed that the prevalence of proteinuria is significantly related to WWI, and WWI (OR=1.28; BMI: OR=1.02; WC: OR=1.01) may be more accurate than other obesity indicators in predicting the incidence rate of proteinuria (40). Wen et al. also validated the predictive efficacy of WWI (WWI: OR=1.33; BMI: OR=1.30; WC: OR=1.28; ABSI: OR=1.21) in infertile populations (14). More importantly, we verified that the correlation between WWI (AUC_WWI_=0.588) and secondary infertility is higher than other typical obesity indicators(WC, BMI, WHtR, WHR, wBMI, BRI and ABSI). In summary, it is widely believed that WWI may be used as a predictive factor for obesity-related diseases, and our research supports this statement.

The relationship between WWI and infertility has been established in recent years, but the mechanism behind this is still unclear, especially in women with secondary infertility. Previous systematic evaluations have shown that obese women have a higher risk of developing depressive symptoms (21), and obesity may lead to changes in the gut microbiota, thereby affecting the host’s emotions and behavior (41). Psychological barriers are becoming increasingly common in modern society. Preexisting emotional disorders, such as anxiety and depression, may hurt the outcome of infertility treatment (42), which may be due to emotional disorders affecting hormone secretion and endometrial growth (20). In the same way, the incidence of psychological depression will significantly increase with the prolongation of infertility, and women with infertility are twice as likely to be affected by emotional disorders as other women (43, 44). Previous studies have shown that depression plays a mediating role in treating and caring for various diseases (45, 46). Based on the large population of NHANES, Pan et al. study showed that the PHQ-9 score was estimated to mediate a 0.2% (P=0.03) association between ABSI and infertility (17). We also found that in secondary infertility women, 3.94% of the relationship between WWI and secondary infertility seems to be mediated by depressive symptoms. These results indicate that improving depression symptoms in patients may reduce the likelihood of secondary infertility caused by WWI.

More and more research is focuses on potential mediators and interventions for reversing infertility, manifested in weight loss, physical activity, dietary factors, and weight loss surgery (47). However, the results of these interventions are not satisfactory. A systematic review shows that cognitive-behavioral treatment(CBT), mind-body interventions(MBI), and stress management skills(SMS) are the most common psychological interventions for infertile women, and are beneficial for improving their mental health status (48). Psychological intervention is equally necessary as medication for infertility (49), and our study suggests the mediating effect of depression between WWI and secondary infertility. Management strategies determined based on female depression scores and WWI may help reduce women’s secondary infertility risk.

Our research has several advantages. Our research is based on national data, and the results are widely applicable to the general population in the United States. Regression analysis was adjusted for covariates, and the large sample size allowed us to conduct sensitivity analysis and subgroup analysis to confirm the robustness of the results. Finally, the mediating role of depression between WWI and secondary infertility was verified. However, there are still some restrictions that need to be declared. Although we have adjusted for some potential covariates, many factors still affect secondary infertility, and we cannot completely rule out the effects of other potential confounding factors, such as other social, environmental and antidepressants use variables. Our research findings are based on one country and race, so it remains to be investigated whether these findings apply to other races or countries. This survey was a cross-sectional study that can only explore the relationship between WWI, depression, and secondary infertility, providing clues for etiological research. It cannot verify the causal relationship between disease and etiology. In addition, in the mediation analysis, depression mediate partly the association between WWI and secondary infertility, but its effect is relatively weak. Therefore, further cohort studies are needed to verify its causal relationship, in order to ensure that readers have a clearer and more accurate understanding of the relationship between depression and secondary infertility in WWI.

## Conclusion

This study shows that WWI and depression are associated with secondary infertility, and WWI’s predictive power for secondary infertility is better than other obesity indicators. In addition, depressive symptoms may play a mediating role in the relationship between WWI and secondary infertility. This suggests that clinical healthcare workers should pay more attention to the mental health of obese women in the future, which may protect them from infertility.

## Data availability statement

Publicly available datasets were analyzed in this study. This data can be found here: www.cdc.gov/nchs/nhanes/.

## Ethics statement

The studies involving humans were approved by The Research Ethics Review Board of the NCHS. The studies were conducted in accordance with the local legislation and institutional requirements. The participants provided their written informed consent to participate in this study.

## Author contributions

FS: Conceptualization, Data curation, Formal analysis, Methodology, Writing – original draft, Writing – review & editing. ML: Conceptualization, Data curation, Formal analysis, Funding acquisition, Supervision, Writing – review & editing. SH: Conceptualization, Data curation, Formal analysis, Funding acquisition, Supervision, Writing – review & editing. RX: Data curation, Formal analysis, Methodology, Software, Supervision, Writing – review & editing. HC: Conceptualization, Formal analysis, Supervision, Writing – review & editing. ZS: Conceptualization, Formal analysis, Supervision, Writing – review & editing. HB: Conceptualization, Formal analysis, Supervision, Writing – review & editing.
